# Appointment keeping for medical review among patients with selected chronic diseases in an urban area of Uganda

**DOI:** 10.11604/pamj.2014.19.229.3971

**Published:** 2014-10-30

**Authors:** Joan Nakayaga Kalyango, Maurice Hall, Charles Karamagi

**Affiliations:** 1 School of Pharmacy, Queens University Belfast, BT9 7BL, Belfast, Northern Ireland; 2 Clinical Epidemiology Unit, Makerere University College of Health Sciences, P.O. Box 7072, Kampala, Uganda; 3 Department of Pharmacy, Makerere University College of Health Sciences, P.O. Box 7072, Kampala, Uganda; 4 Department of Paediatrics and Child health, Makerere University College of Health Sciences, P.O. Box 7072, Kampala, Uganda

**Keywords:** Chronic disease, medical review appointments, missed appointments

## Abstract

**Introduction:**

Proper management of chronic diseases is important for prevention of disease complications and yet some patients miss appointments for medical review thereby missing the opportunity for proper monitoring of their disease conditions. There is limited information on missed appointments among chronic disease patients in resource limited settings. This study aimed to determine the prevalence of missed appointments for medical review and associated factors among chronic disease patients in an urban area of Uganda.

**Methods:**

Patients or caregivers of children with chronic diseases were identified as they bought medicines from a community pharmacy. They were visited at home to access their medical documents and those whose chronic disease status was ascertained were enrolled. The data was collected using: questionnaires, review of medical documents, and in-depth interviews with chronic disease patients.

**Results:**

The prevalence of missed appointments was 42% (95%CI = 35-49%). The factors associated with missed appointments were: monthly income ≤30US Dollars (OR = 2.56, CI = 1.25–5.26), affording less than half of prescribed drugs (OR = 3.92, CI = 1.64–9.40), not experiencing adverse events (OR = 2.66, CI = 1.26–5.61), not sure if treatment helps (OR = 2.84, CI = 1.047.77), not having a medicines administration schedule (OR = 6.77, CI = 2.11–21.68), and increasing number of drugs (OR = 0.72, CI = 0.53–0.98).

**Conclusion:**

Patients missed appointments mainly due to: financial and health system barriers, conflicting commitments with appointments, and perceptions of the disease condition. Patients should be supported with accessible and affordable health services.

## Introduction

The global prevalence of chronic diseases is rapidly increasing and developing countries are expected to experience the greatest brunt of this rise [1]. Most of these diseases if not properly managed are associated with complications leading to increased morbidity and mortality [2]. The proper management of most chronic diseases usually involves drugs or other non-pharmacologic interventions. However, response to these disease management strategies varies from person to person and may even vary in the same person over time. In addition, some patients may experience adverse events or self-care challenges that predispose them to non-adherence [3] resulting in poor treatment outcomes. In resource limited settings without access to free medicines and services, patients may not be able to afford drugs and other necessities for management of their disease conditions [4, 5]. It is therefore important that patients are monitored regularly by their health provider to identify the impediments to treatment that patients may be experiencing and adjust treatment where necessary [6].

The opportunity for proper patient assessment is usually presented when patients go to their health providers for medical reviews. The important starting point of this process is when patients keep their appointments for medical review. Patients that miss their appointments with health providers create delays or lack of monitoring of their conditions predisposing them to exacerbations of disease and diseases-related complications. They are likely to end up admitted or making emergency consultations because of poor disease outcomes [7–10]. This increases the burden on the health system as well as loss of productivity due to illness. Studies done elsewhere have reported magnitudes of missed appointments at 10-42% [11–13] and the factors that have been related to non-attendance of clinics include: distance to the health facility, lifestyle characteristics like drinking and smoking, conflicts with the clinician on how the disease should be managed, forgetfulness, transportation difficulties, work schedules, perceptions of benefit from the review, perceived disrespect, not understanding appointment scheduling system, age, long waiting times and duration of illness [7, 9, 14, 15]. In Uganda, the magnitude and factors associated with missed medical review appointments among chronic disease patients are unknown and yet the burden of chronic diseases is on the increase [16]. The aim of this study therefore was to determine the prevalence of missed appointments for medical review and associated factors among chronic disease patients seeking medicines from a community pharmacy so as to identify strategies that can be used to improve care of chronic disease patients.

## Methods

### Study design and setting

A mixed methods study with qualitative and quantitative methods of data collection was conducted from June to July 2010. The study was conducted in Kalerwe which is found in Kawempe division in Kampala district, the capital city of Uganda. Kalerwe is an urban area that is both residential and commercial with small scale trade being the main commercial activity. At the time of the study, the area was served by three community pharmacies but some of the clients who visit these pharmacies come from places that are outside Kalerwe. The pharmacy from which the study participants were identified is within a distance of about one kilometre from Mulago hospital, a National referral and teaching hospital in Uganda and this hospital serves as one of the main sources of clients for the pharmacy.

### Study participants

The study enrolled chronic disease patients that had been on treatment for at least two months, lived within one kilometre of the pharmacy, and gave informed consent to participate in the study. In cases where the patient was a child, the primary caregiver was enrolled as the respondent. The chronic diseases that were considered for inclusion into the study were: asthma, chronic obstructive pulmonary disease, cardiovascular diseases (angina, congestive heart failure, hypertension, stroke), arthritis, thyroid diseases, diabetes mellitus, chronic hepatic disorders, chronic kidney disorders, hyperlipidemias, sickle cell disease and gastro-intestinal diseases.

### Sampling and sample size estimation

One of the three community pharmacies in Kalerwe area was purposively chosen because it had the biggest clientele. The selected community pharmacy was used as the point at which the chronic disease patients could be identified because of the nature of the research question. In order to identify chronic disease patients that had missed their medical reviews, it was necessary to use a place where even those patients that had missed their appointments could be found. This was because at medical outpatients of hospitals or clinics one was likely to miss the patients that do not present to health facilities for long time. The record system for appointments for most of the health facilities is not sufficient to capture patients that have missed medical review and some patients change their health providers without informing the facility where they have been seeking care. It is common for patients to continue buying medicines using old prescriptions because patients in Uganda can access drugs without presentation of a valid current prescription. A total of 207 participants were consecutively enrolled but three subjects were excluded due to missing information on appointment dates given for medical review. The outcome among these patients could therefore not be determined. The sample size of 207 was estimated using the Kish Leslie formula [17], assuming missed appointments in 42% of patients [11] and adjustment for having an expected population of about 450 chronic disease patients during the study period.

### Data collection

The quantitative data was collected by research assistants that had been trained about the study, supervised by the first author (JNK). The patients were informed about the study at the pharmacy and those that met the selection criteria were visited at home by a member of the research team in order to access their medical documents. An interviewer administered questionnaire translated into the main local language spoken in the area (Luganda) was used to collect data on participants’ socio-demographic characteristics; household characteristics; drug- and disease-related characteristics; health facility-related characteristics; knowledge and perception of the disease; drugs, and health care received; lifestyle characteristics; and home management of medicines [18]. The patients’ medical forms were reviewed to confirm information about the disease, prescribed drugs, the last date of review by health provider and the date for the next appointment.

#### Variables definition

Missed medical review appointment was defined as not presenting to the health provider for a period longer than the last appointment date. The qualitative data was collected using in-depth interviews with chronic disease patients that had not taken part in the quantitative part of the study and its purpose was to explain the results from the quantitative data. Ten in-depth interviews were conducted and the participants were selected purposively. A topic guide was used to direct the interviews and the proceedings of the interviews were tape recorded.

### Data management and analysis

The quantitative data was captured in Epidata version 2.1b (The EpiData Association, Odense, Denmark) and was exported to STATA 10 (Stata, College Station, TX, USA) for analysis. Descriptive statistics were used to describe the characteristics of study participants and their appointment keeping for medical review. Unadjusted and adjusted analyses were done using logistic regression to determine the factors that were associated with appointment keeping. The adjusted analysis included factors that had p-values less than 0.2 at unadjusted analyses and it involved identification of independent predictors of missed appointments as well as checking for interaction and confounding. Interaction was assessed using the chunk test that compared full and reduced models. Confounding was assessed by comparing unadjusted and adjusted odds ratios and was considered present if a variable changed the odds ratio of another by more than 10%. The qualitative data was transcribed and translated into English. It was analyzed manually using content analysis to generate codes and categories [19]. The transcripts were read several times to identify meaning units which were used to generate codes. The codes were subsequently used to generate categories.

### Ethical approval

The study was approved by the School of Medicine Research and Ethics Committee and the Uganda National Council of Science and Technology (HS783).

## Results

### Socio-demographic and disease characteristics of the participants

About 84% (n = 171) of the participants were chronic disease patients while the rest were primary caretakers. The majority were females (67%, n = 137) and half were married (50%, n = 99). The most common occupation was businessperson (38%, n = 77) while the most common education level attained was secondary school (34%, n = 67). The most common disease was cardiovascular disease (43%) followed by diabetes (26%). The mean age of the participants was 49 (SD = 17.1) years while their median monthly income was equivalent to 125 US dollars. The median number of chronic diseases suffered by the participants was one (minimum = 1, maximum = 4) and the mean number of prescribed drugs was three (SD = 1.2) (Table 1).

**Table 1:** Socio-demographic and clinical characteristics of 204 chronic disease patients in an urban area in Uganda

Characteristic	Measure
Female sex (N = 204), n(%)	137 (67.2)
**Marital status (N = 199), n(%)**	
Married	99(49.8)
Single	40 (20.1)
Separated	17 (8.5)
Widowed	43 (21.6)
**Religion (N = 202), n (%)**	
Catholic	59 (29.2)
Anglican	54 (26.7)
Muslim	61 (30.2)
Pentecostal	23 (11.4)
Other*	5 (2.5)
**Occupation (N = 203), n (%)**	
Student	10 (4.9)
Housewife	22 (10.8)
Businessperson	74 (36.5)
Salaried employee	36 (17.7)
Unemployed	50 (24.6)
Other^	11 (5.4)
**Highest education attained (N = 198), n (%)**	
None	27 (13.6)
Primary	55 (27.8)
Secondary	67 (33.8)
Tertiary	49 (24.8)
**Disease (N = 202), n (%)**	
Cardiovascular	87 (43.1)
Diabetes	53 (26.2)
Asthma	30 (14.9)
Arthritis	22 (10.9)
Sickle cell disease	18 (8.9)
Others†	28 (13.9)
Mean age (SD)	49 (16.9)
Median monthly income in USD# (min, max)	125 (0, 4000)
Median number of diseases (min, max)	1 (1, 4)
Mean number of prescribed drugs (SD)	3 (1.2)

* Includes Seventh Day Adventists and Jehovah's witness

^ Includes preachers, cobbler, farmer

† Includes thyroid and gastrointestinal diseases

# United States Dollars

### Missed appointments for medical review

The quantitative results on appointment keeping are summarized in Table 2. About 42% (n = 86, 95% CI =35-49%) of the patients missed their appointments with the health provider. The most common reason given for missing appointments was feeling well (40%, n = 34) followed by having no money for transport (30%, n = 26). These reasons were also given for missing appointments from the qualitative data.

**Table 2:** Appointment keeping among 204 chronic disease patients in an urban area in Uganda

Variable	Frequency	Percent
Missed appointment (N = 204)	86	42.2
Reasons for missing appointment (N = 86)		
Was feeling well	34	39.5
Had no money for transport	26	30.2
Had no money for services	15	17.4
Was busy	13	15.1
I forgot	12	14.0
I know my drugs	6	7.0
Was not told return date	3	3.5
Didn't find the health worker	2	2.3
Other reason*	7	8.1

* Include switched to herbs, medicines not working, tired of going to facility, legs are painful and could not walk, blind and had no one to assist, child had gone for holiday

“I feel fine and yet I am too busy so I do not see the point”, said one KI. “At times I have no money for transport so I cannot go”, said another KI.

Other reasons for missing appointments from the qualitative findings included health system barriers and conflicting appointment schedules with work as shown by the quotes below:

“The waiting queue is too long so I waste a lot of time at the doctor's. Sometimes I am even discouraged from going there because of this”, said one male KI. “Sometimes I have to travel upcountry and at work they don't consider such issues. For as long as one can walk then one has to work. Yet it is hard to make another appointment since I go to a government hospital”, said a female KI.”

The median time since the last visit to the health provider among those that had missed their appointments was 2.3 months (minimum 4 days, maximum 6 years).

### Factors associated with missed appointments

The factors that had p-values less than 0.2 at unadjusted analysis were: sex, religion, income, who pays for treatment, proportion of drugs patients could usually afford, experience adverse events, knowledge of drugs, feel treatment helps, smoke, take alcohol, distance to the health unit, have medicines administration schedule, and number of drugs prescribed at the last visit to the health provider. When these were used in adjusted analysis, the factors that remained significantly associated with missed appointments were: income =30USD per month (OR = 2.56, CI = 1.25 – 5.26), affording less than half of prescribed drugs (OR = 3.92, CI = 1.64 – 9.40), not experiencing adverse events (OR = 2.66, CI = 1.26 – 5.61), not sure if treatment helps (OR = 2.84, CI = 1.04 – 7.77), not having medicines administration schedule (OR = 6.77, CI = 2.11 – 21.68), and increasing number of drugs (OR = 0.72, CI = 0.53 – 0.98). Unadjusted and adjusted analyses are presented in Table 3.

**Table 3:** Factors associated with missed appointments at unadjusted and adjusted analysis

Variable	Not kept Appointment n (%)	Unadjusted OR (95% CI)	P-value	Adjusted OR (95%CI)	P-value
**Sex**					
Male	22 (32.8)	1.00			
Female	64 (46.7)	1.79(0.97-3.30)	0.06		
**Religion**					
Catholic	19 (32.2)	1.00			
Anglican	26 (48.2)	1.95 (0.91-4.20)	0.09		
Muslim	25 (41.0)	1.46 (0.69-3.09)	0.32		
Pentecostal / Other	15 (53.6)	2.43 (0.97-6.11)	0.06		
**Income**					0.01
≤ 30US dollars*	47 (55.3)	1.00		1.00	
> 30US dollars	30 (31.2)	0.37 (0.20-0.67)	0.001	0.39 (0.19-0.80)	
**Pays for treatment**					
Self	39 (35.1)	1.00			
Other	46 (50.0)	1.83 (1.05-3.25)	0.03		
**Proportion of drugs afforded**					0.002
Half or above	58 (35.6)	1.00		1.00	
Less than half	27 (67.5)	3.76 (1.80-7.84)	<0.001	3.92 (1.64-9.40)	
**Adverse reactions**					0.01
Yes	26 (32.5)	1.00		1.00	
No	60 (48.4)	1.95 (1.08-3.50)	0.03	2.66 (1.26-5.61)	
**Knowledge of drugs**					
Yes	48 (36.9)	1.00			
No	38 (51.3)	1.80 (1.01-3.22)	0.05		
**Feel treatment helps**					0.04
Yes	64 (38.1)	1.00		1.00	
No / Not sure	22 (61.1)	2.55 (1.22-5.35)	0.01	2.84 (1.04-7.77)	
**Smoke**					
Yes	5 (71.4)	1.00			
No	80 (40.8)	0.25 (0.05-1.26)	0.09		
**Take alcohol**					
Yes	9 (23.7)	1.00			
No	76 (46.1)	2.75 (1.23-6.17)	0.01		
**Distance to health unit**					
≤ 5 km	68 (46.3)	1.00			
> 5 km	15 (32.6)	0.56 (0.28-1.13)	0.11		
**Have administration schedule**					0.001
Yes	64 (36.8)	1.00		1.00	
No	22 (73.3)	4.73 (1.99-11.24)	<0.001	6.77 (2.11-21.68)	
Number of drugs		0.83 (0.64-1.06)	0.13	0.72 (0.53-0.98)	0.04

* Used level that defines poverty (1 dollar a day)

Age, education level, marital status, household characteristics, number and duration of diseases, perceived severity of disease, knowledge of disease, get adequate information about the disease, and type of health facility had p-values higher than 0.2 at unadjusted analysis and were therefore not considered at adjusted analysis.

## Discussion

In this study about 4 in every 10 patients missed their appointments with the health care provider. Most patients missed their appointments because they felt well or they had no money for transport or services. The factors significantly associated with missed appointments were: higher income (OR = 0.39), non affordability of medicines (OR = 3.92), not experiencing adverse events (OR = 2.66), not sure if treatment helps (OR = 2.84), lack of medicines administration schedule (OR = 6.77), and increasing number of drugs (OR = 0.72). Appointment keeping for medical review among these patients was low yet this is an important aspect of chronic disease management. Patients need to be continuously evaluated for worsening of disease, experiencing of adverse effects or even improvements in condition, all of which may necessitate altering the patient's medication. The initial step that is necessary for this to happen is that the patient goes to their health provider for review. The proportion of patients in this study that did not keep their appointments was higher than that found in a study in a Gastroenterology unit in Northern Ireland in which non-attendance at clinics ranged from 10 – 25% [12] and that in a study done in Saudi Arabia in which non-attendance was 30% [20]. However, it was similar to the average rate of non-compliance with appointments (42%) reported in a review article [11].

The most common reasons given for missing appointments (feeling well and having no money for transport) were also reflected in the qualitative findings. They were however slightly different from those in the studies in Northern Ireland [12] and Saudi Arabia [20]. In the Northern Ireland study, most patients that missed appointments did so because they had forgotten and only 8% missed because they felt better and in the Saudi Arabia study, forgetfulness and lack of transport were the most common reasons given.

Patients that could afford less than half of prescribed medicines were more likely to miss their appointments. Patients that cannot afford all their medicines may also experience challenges in keeping their appointments because they do not have money for transport or for services. In a study done among patients in a community health centre in Massachusetts, USA, patients that did not have insurance coverage and therefore had to pay for their treatment out-of-pocket were more likely to miss appointments [21].

Chronic disease patients that did not experience adverse affects were more likely to miss their appointments compared to those that experienced adverse effects. Patients that do not experience adverse effects may not feel the need to see their health provider especially if they feel they are responding to treatment. A systematic review revealed that patients that perceived that they had no need for an appointment were more likely to miss it [14]. From the qualitative findings, patients sometimes missed appointments because they felt fine and did not see the need to go for review.

Lack of a medicines administration schedule was associated with higher odds of missing appointments. Patients that do not have a medicines administration schedule are likely to be non-adherent to medicines. Non-adherence to medicines has been associated with non-adherence to other disease management strategies e.g. keeping medical review appointments [22]. Patients that lack medicines administration schedules may have hectic lifestyles that lack the structure that is necessary to keep appointments or to establish a routine in which they take medicines. It is also possible that patients who miss their appointments do not get the necessary health education needed from their health providers and therefore do not take their medicines as required.

In this study, the odds of missing appointments reduced with increasing number of drugs prescribed for the patient. Patients with more drugs may have more severe disease or they may perceive their disease as more severe compared to those with fewer drugs and are therefore motivated to keep their appointments. According to the health belief model, patients are more likely to take a health action if they perceive themselves to be at risk of negative consequences [23]. However, perceived severity of illness was not significant in this study.

Lower income was associated with higher odds of missed appointments. Patients with more income may have fewer hindrances to keeping their appointments compared to those with less income. They are less likely to miss appointments due to lack of transport or money for services. This is supported by the qualitative findings that some patients miss their appointments due to lack of money for transport. The findings are different from those in study done in Saudi Arabia where there was no association between income and keeping appointments [20]. This difference could be due to the fact that the patients in the study in Saudi Arabia the patients were all from a public hospital where services are provided free whereas in our study some of the patients attended private hospitals. However, low gross income has been reported as one of the factors associated with non-attendance at clinics in a systematic review [14].

Patients that were not sure if their treatment helps were more likely to miss their appointments compared to those who felt the treatment helps. If patients do not feel the benefit from taking their medicines, they may not go back to the health provider. In a systematic review by Paterson and others, patients who lacked confidence that the appointment would be of benefit were less likely to attend the clinic appointments [14].

Sex, age, religion, education level, marital status, number of people per room, number of diseases, duration of disease, perceived severity of illness, knowledge of disease, adequacy of information received, smoking, distance to and type of health facility, appropriate storage practices, and appropriate medication management practices were not significantly associated with keeping appointments both at bivariate and multivariate analysis. Occupation, who pays for the treatment, knowledge of drugs, and alcohol consumption were significant at unadjusted but not at adjusted analysis. Some of the findings are in contrast to those in other studies done in Massachusetts and Saudi Arabia that reported age and gender to be associated with appointment keeping [21, 24]. The study in Massachusetts evaluated appointment keeping over a 12-month period while that in Saudi Arabia was done in a general clinic and patients did not necessarily have chronic diseases.

### Study limitations

This study may not have had adequate power to detect associations for some of the variables that have been reported in the literature to be related to medicines use monitoring. The sample size computation used was primarily based on prevalence of missed appointments and thus may have been insufficient to assess many of the associations.

## Conclusion

The magnitude of missed appointments for medical review was quite high implying that patients with chronic disease are at risk of disease complications through non-monitoring of their disease conditions. The patients were mainly hindered from keeping appointments by financial barriers, health system barriers, conflicting commitments with appointments and perceptions about their disease or treatment. Chronic disease patients therefore need to be supported with easily accessible and affordable high quality health services with adequate communication with the health providers.
